# Radioprotective Effect of ε-Aminocaproic Acid in Acute Total-Body Gamma Irradiation in Rats

**DOI:** 10.3390/life16010096

**Published:** 2026-01-08

**Authors:** Timur Fazylov, Timur Saliev, Igor Danko, Zhomart Beksultanov, Shynar Tanabayeva, Ildar Fakhradiyev, Anel Ibrayeva, Marat Shoranov

**Affiliations:** 1 Department of Medicine, S.D. Asfendiyarov Kazakh National Medical University, Almaty 050000, Kazakhstan; timson1193@mail.ru (T.F.);; 2 Institute of Nuclear Physics, Almaty 050032, Kazakhstan; 3 College of Medicine, Korea University, Seoul 02841, Republic of Korea

**Keywords:** ε-aminocaproic acid, radioprotection, acute radiation syndrome, intestinal mucosal injury, total-body irradiation

## Abstract

Background. Acute radiation injury to the small-intestinal mucosa and the hematopoietic system is a key determinant of early mortality after high-dose total-body irradiation. ε-Aminocaproic acid (EACA), a lysine analogue with antifibrinolytic properties, has been proposed as a potential radioprotective agent, but its effects on intestinal and hematologic injury remain insufficiently characterized. Methods. In this experimental study, 240 male Wistar rats were subjected to single-dose total-body γ-irradiation at 10.6 Gy and randomized into six groups: two non-irradiated controls (CG-1, CG-2), an irradiated control without treatment (CG-3), and three experimental groups receiving EACA (EG-1: 3 h before irradiation; EG-2: 3 h after irradiation; EG-3: both 3 h before and 3 h after irradiation). Pain behavior was assessed using the Rat Grimace Scale. Intestinal damage was evaluated by a modified Radiation Injury Intestinal Mucosal Damage Score (RIIMS_sum), villus and crypt morphometry, and qualitative histology of the ileum. Hemoglobin, leukocytes, and platelets were measured serially, and 30-day survival was analyzed using Kaplan–Meier curves with log-rank tests. Results. Across all EACA regimens, the odds of being in a higher Rat Grimace Scale pain category were reduced compared with CG-3, with the strongest effect in EG-3 (OR 0.42; 95% CI 0.31–0.58). At 72 h after irradiation, the cumulative RIIMS score was lower in EACA-treated groups by approximately 17–36% versus CG-3, with the lowest injury in EG-3 (18.5 vs. 29.0 points). EACA attenuated shortening and blunting of villi, preserved crypt architecture, and mitigated anemia, leukopenia, and thrombocytopenia. Thirty-day survival was 20% in CG-3 and 60%, 65%, and 80% in EG-1, EG-2, and EG-3, respectively (all *p* < 0.05 vs. CG-3). Conclusions. ε-Aminocaproic acid exerts a pronounced, timing-dependent radioprotective effect in a rat model of acute total-body γ-irradiation, concurrently reducing the severity of radiation enteritis, hematologic toxicity, and early mortality. These findings support further investigation of EACA as a candidate adjunct in the prevention of acute radiation injury.

## 1. Introduction

Effective protection against ionizing radiation remains one of the key challenges in modern medicine and radiobiology, as it concerns both patients undergoing diagnostic procedures with externally generated X-rays (e.g., CT) and professionals exposed to γ-irradiation from radioisotope sources or bremsstrahlung [1], as well as workers who routinely handle ionizing-radiation sources, including nuclear power plant personnel [2]. In clinical practice, ‘gamma irradiation’ spans (i) diagnostic nuclear-medicine procedures (SPECT/PET) that use trace activities of radionuclides (e.g., 99mTc, ^123^I, ^18^F) and deliver effective doses typically in the millisievert range (≈2–10 mSv per study), and (ii) therapeutic/external exposures such as cobalt-60/cesium-137 teletherapy or bremsstrahlung-based total-body irradiation (TBI) that deliver organ/tumor doses in grays (≈1.8–2.0 Gy per fraction in conventional RT; TBI cumulatively ~2–12 Gy). Our study models the latter scenario—high-dose total-body γ-exposure (10.6 Gy single fraction from bremsstrahlung on a tantalum converter), not diagnostic imaging—to interrogate acute intestinal and hematopoietic injury. The intestine and the hematopoietic system are the most vulnerable to high-dose total-body irradiation, forming the two main components of acute radiation syndrome [3,4,5]. Damage to crypt stem cells, loss of mucosal barrier function, and translocation of microbiota and proinflammatory mediators lead to a severe gastrointestinal syndrome with pronounced diarrhea, sepsis, and multiple organ failure, while suppression of hematopoiesis, bone marrow aplasia, and profound cytopenia underlie the hematopoietic variant [6]. Experimental and clinical data emphasize that the intestine is one of the most vulnerable organs under total-body and abdominal irradiation, and that the volume and dose of radiation exposure are closely associated with the risk of severe complications [7,8]. The combined impact on the intestinal and hematopoietic components largely determines early mortality and depends on the total dose and volume of irradiated tissue, as well as on the rate of development of infectious and hemorrhagic complications [9].

In recent decades, a wide range of experimental radioprotectors has been described, including small molecules and biological agents aimed at reducing apoptosis, suppressing oxidative stress, and modulating the inflammatory response. It has been shown that certain compounds, such as Ex-RAD^®^ (ON01210.Na), in a mouse model of total-body irradiation, attenuate hematopoietic and intestinal injury, increase the dose-reduction factor, and accelerate hematopoietic recovery [10]. These data support the fundamental feasibility of pharmacologic modification of radiation damage to critical target organs.

Despite the clear need for safe and effective radioprotectors, only a few agents with radioprotective or cytoprotective properties have been approved at the clinical level, primarily amifostine and palifermin [11,12]. Their use is limited by narrow indications and toxicity profiles, and the achieved effect does not provide complete prevention of damage caused by ionizing radiation, especially in the setting of total-body irradiation with the development of gastrointestinal and hematopoietic syndromes.

In addition to clinically used agents, a number of systemic radioprotectors are being actively investigated in preclinical models. In particular, it has been shown that granulocyte colony-stimulating factor (G-CSF), traditionally regarded as a drug for correcting the hematologic syndrome, can also exert protective effects against radiation-induced intestinal injury: in experimental studies, G-CSF reduced apoptosis of crypt cells and attenuated the severity of structural alterations and inflammatory infiltration of the mucosa [13].

A promising avenue also remains the search for natural compounds with radioprotective activity. At the preclinical stage, various low-molecular-weight antioxidants are being investigated, including organic acids and polyphenols [14,15]. Their effects are thought to be mediated through reduced generation of reactive oxygen species, activation of endogenous antioxidant systems, stabilization of cellular homeostasis, and modulation of the immune response [16]. Nevertheless, even the most promising antioxidants currently do not provide reliable whole-body radioprotection.

In this context, particular interest is given to agents with a well-characterized safety profile that are already used in clinical practice and are capable of modulating the proteolytic and inflammatory cascades involved in the response to radiation injury. ε-Aminocaproic acid (EACA) is a well-known fibrinolysis inhibitor that is widely used in clinical practice as a hemostatic agent [17]. A number of experimental studies have shown that EACA can prevent or reduce radiation-induced damage, which makes it a potential candidate for radioprotective therapy and justifies the need for more in-depth investigation of its effects [18,19]. However, detailed in vivo studies evaluating the radioprotective effects of EACA under combined pre- and post-irradiation administration, particularly with respect to the intestine and hematopoiesis, are lacking.

In this regard, the aim of the present study was to evaluate the radioprotective properties of ε-aminocaproic acid in an experimental model of acute total-body gamma irradiation in rats, analyzing its effects on survival, the severity of clinical manifestations, and morphological changes in the intestinal mucosa and hematopoietic system under different administration regimens.

## 2. Materials and Methods

### 2.1. Ethical Issues

The protocol of the experimental study was approved by the Local Ethical Committee of the S.D. Asfendiyarov Kazakh National Medical University (approval No. 24 (160) of 28 January 2025).

### 2.2. Study Design

For the in vivo studies, 240 male Wistar rats, 6–8 weeks old, weighing 180–200 g at baseline were obtained from the vivarium of the Scientific Research Institute named after B. Atchabarov. All animals were housed under standard pathogen-free conditions, maintained at a room temperature of 15–25 °C, with 50–60% relative humidity, and a 12 h light/dark cycle. The rats were provided with free access to food and water.

**Figure 1:**
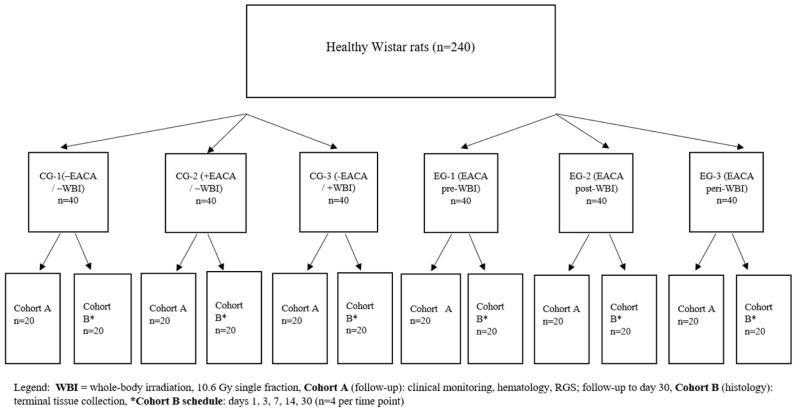
Flowchart of experimental groups.

Laboratory animals were randomly divided into 6 groups:

(1) control group (CG-1) (no drug/no exposure) 40 (rats);
(2) control group (CG-2) (drug/no exposure) 40 (rats);
(3) control group (CG-3) (no drug/exposure) 40 (rats);
(4) experimental group (EG-1) (drug before 3 h exposure) 40 (rats);
(5) experimental group (EG-2) (drug 3 h after exposure) 40 (rats);
(6) experimental group (EG-3) (drug 3 h before and after 3 h exposure) 40 (rats) (Figure 1).

In all groups, the timing of procedures was standardized. Animals that did not receive EACA (CG-1 and CG-3) were given an oral administration of an equivalent volume of sterile water via gavage at the corresponding time points, mimicking the drug administration procedure. Non-irradiated rats (CG-1 and CG-2) were placed in the same containers and transported to the irradiation area, where they were subjected to a sham procedure (fixation and rotation of the container without turning on the radiation), thereby equalizing stress and procedure duration across groups.

A total of 40 animals were included in each of the six groups. To avoid cross-influence on outcomes, each group was pre-divided equally into two independent cohorts: cohort A (survival, pain, hematology; n = 20 per group) and cohort B (cross-sectional histology; n = 20 per group).

### 2.3. Cohorts and Sampling Schedule

Animals in cohort A were used to assess 30-day survival and to perform longitudinal recording of pain and hematologic parameters. Grimace scoring and blood sampling were carried out on days 1, 3, 7, 14, and 30 of follow-up. In cohort A, no tissue sampling for morphological analysis was performed. Overall mortality within 30 days was recorded; for non-irradiated groups, any deaths were classified by veterinary specialists as non-radiation-related and still counted as events. Animals in cohort B were used exclusively for cross-sectional morphological and morphometric assessments of the ileum. Euthanasia was performed on days 1, 3, 7, 14, and 30; at each time point, 4 rats per group were withdrawn from the experiment, providing n = 4 animals/group/time point for morphological analysis. For the analysis of 30-day survival, observation time from both cohort A and cohort B was taken into account: animals in cohort B contributed to the risk set up to the pre-specified time of euthanasia, after which their observations were treated as right censored. Thus, Kaplan–Meier curves were constructed based on all 40 animals in each group.

Pain grimace scoring and histological evaluation were performed by two independent, blinded assessors. Operators who performed irradiation could not, for obvious reasons, be blinded to the fact of exposure, but they did not participate in outcome assessment or data analysis.

### 2.4. Radiation Exposure

The irradiation of animals was carried out at the Institute of Nuclear Physics (Kazakhstan, Almaty). The radiation treatment was performed using ILU-10 accelerators (Budker Institute of Nuclear Physics, Russian Federation). Rats were subjected to whole-body irradiation (WBI) at a dose of 10.6 Gy; this dose was selected based on the results of previous studies [20,21].

Irradiation was performed using an ILU-10 electron accelerator with bremsstrahlung radiation generated on a tantalum converter. The distance from the converter to the surface of the transport table was 350 mm. An absorbed dose of 10.6 Gy was delivered in a single fraction at an average dose rate of approximately 2.5 Gy/min over 4 min (calculated from EPR dosimetry data). For dosimetric control, six alanine dosimeters were used, and measurements were performed on an ESP 300E EPR spectrometer (Bruker, Karlsruhe, Germany); dose field non-uniformity along the length of the table did not exceed ±10%. This whole-body single-fraction dose was intentionally selected to model a severe acute radiation syndrome scenario rather than routine diagnostic exposures or conventional radiotherapy regimens.

Before irradiation, the animals were sedated with intraperitoneal pentobarbital (40 mg/kg body weight). During irradiation, the container was continuously rotated in front of the radiation source to ensure uniform exposure. The animals were then returned to their cages and provided with food and water.

### 2.5. Design of the In Vivo Study

The experiments were carried out at the Research Institute named after B. Atchabarov, at the Laboratory of Experimental Medicine. EACA powder (Sigma-Aldrich, St. Louis, MO, USA) was dissolved in sterile water to 25 mg/mL and administered orally by gavage at 0.45 g/kg (dose volume adjusted to body weight). Water was selected as a physiologically neutral vehicle commonly used for oral dosing in rodents.

### 2.6. Animals’ Pain Assessment

Grimace pain scores (Rat Grimace Scale) [22] (ordinal 0–2) were summarized as median (IQR). Group differences over time were analysed using a cumulative link mixed model (CLMM, logit link) with fixed effects for group, day (categorical), and group × day, and random intercepts for rat (repeated measures) and rater. Proportional-odds assumptions were examined, where violated, category-specific effects were fitted in sensitivity analyses. We report proportional-odds ratios (ORs) with 95% CIs. As sensitivity analyses, we used ordinal GEE (cumulative logit, exchangeable working correlation) and non-parametric tests (Kruskal–Wallis with Dunn–Holm post hoc) at each day. Inter-rater reliability was assessed using quadratic-weighted Cohen’s κ and ICC(2,1). Two-sided α = 0.05 with Holm adjustment for multiple comparisons.

### 2.7. Histological Evaluation and Measurement of the Height of the Villi

Ileal samples were collected at distances of 5 mm and 20 mm from the ileocecal junction, rinsed with isotonic saline, and fixed in 10% buffered formalin for 24 h. Tissues were then processed using standard paraffin embedding protocols, sectioned at 5 μm thickness, and stained with hematoxylin and eosin (H&E) for morphological evaluation.

Histological slides were examined using a Leica DMI6000 (Leica, Wetzlar, Germany) light microscope at magnifications ranging from ×100 to ×400. Images were captured with a digital camera integrated into the microscope system. Morphological analysis was carried out in a blinded manner by two independent observers.

Quantitative and semi-quantitative assessment of small intestinal mucosal injury was performed using the modified RIIMS (Radiation Injury Intestinal Mucosal Damage Score) scale with calculation of the total damage index (RIIMS_sum) [21]. Parameters included: goblet cell density, villus length and morphology, crypt cellularity and regeneration, nuclear atypia, lymphatic congestion, mucosal necrosis and exfoliation, inflammation and edema. For domains reflecting changes in cell numbers or structural dimensions, the percentage thresholds of the original scale were used; in addition, signs of repair and inflammatory response were recorded.

For the morphometric analysis, digital software (Image-Pro plus 6.0; Media Cybernetics, Rockville, MD, USA) was used. The height of the villi was measured from the apex to the base of the villus, while the depth of the crypt was measured from the base of the villi to the mucosa. For each indicator, the average height and depth were calculated by measuring 10 times randomly selected villi and crypts. The ratio of villi to crypt was determined by dividing villus height by crypt depth.

### 2.8. Hematologic Assessments

Hemoglobin, leukocytes, and platelets were measured serially in the same animals on days 1, 3, 7, 14, and 30 post-irradiation. Blood was collected in the morning from the tail vein into EDTA (K2) tubes, with stress factors minimized and anesthesia administered according to the institutional protocol; samples were analyzed on the same day on an automated hematology analyzer “Mindray BC-2800, Mindray, China” (class 3-diff; daily internal quality control and calibration according to the manufacturer’s instructions).

### 2.9. Statistical Analysis

Statistical analysis was performed using IBM SPSS Statistics for Windows, Version 25.0 (IBM Corp., Armonk, NY, USA). Sample size was calculated using G*Power 3.1 with the following parameters: effect size (f) = 0.25, statistical power (1 − β) = 0.80, and significance level (α) = 0.05. Numerical data (e.g., villus height, crypt depth, hematologic parameters) were presented as mean ± standard deviation (SD). Between-group differences for continuous variables were analysed using one-way or two-way analysis of variance (ANOVA) with Tukey’s HSD post hoc test for multiple comparisons (post hoc procedures were applied only when the global ANOVA *p*-value was <0.05).

Semi-quantitative RIIMS scores (RIIMS_sum) were treated as ordinal data, summarized as median (interquartile range, IQR) for each group and time point, and compared between groups using the Kruskal–Wallis test with Dunn–Holm post hoc adjustment for multiple pairwise comparisons. Ordinal Rat Grimace Scale (RGS) scores (0–2) were also presented as median (IQR) and analysed using a cumulative link mixed model with fixed effects for group, day, and their interaction, and random intercepts for animal and rater. In sensitivity analyses, we additionally applied generalized estimating equations for ordinal outcomes and non-parametric Kruskal–Wallis tests with Dunn–Holm correction for pairwise comparisons at individual time points.

Survival was evaluated using Kaplan–Meier curves and compared between groups with the log-rank test. In all analyses, two-sided *p*-values < 0.05 were considered statistically significant.

## 3. Results

### 3.1. Assessment of Pain on the RGS

Pooled odds ratios for being in a higher RGS pain category in the experimental groups compared with the irradiated control (CG-3) are presented in Table 1. In all EACA administration regimens, there was a significant reduction in the odds of being classified into a higher pain category compared with CG-3 (EG-1: OR 0.72; EG-2: 0.56; EG-3: 0.42; all *p* ≤ 0.016). The obtained OR values demonstrate a clear effect gradient (EG-3 > EG-2 > EG-1) and confirm that combined drug administration before and after irradiation provides the greatest reduction in pain severity. Inter-rater agreement between the two observers was high (κ_w = 0.83; ICC(2,1) = 0.89), indicating good reproducibility of the RGS in this protocol.

### 3.2. Semi-Quantitative Mucosal Injury Index (RIIMS)

**Figure 2:**
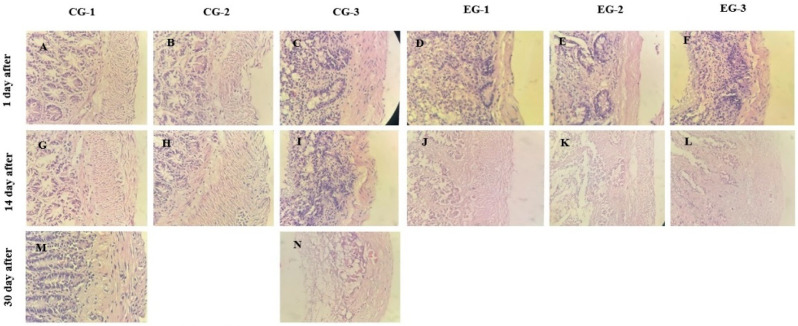
Dynamics of morphological changes in the small intestinal mucosa following irradiation: (**A**) CG-1, day 1; (**B**) CG-2, day 1; (**C**) CG-3, day 1; (**D**) EG-1, day 1; (**E**) EG-2, day 1; (**F**) EG-3, day 1; (**G**) CG-1, day 14; (**H**) CG-2, day 14; (**I**) CG-3, day 14; (**J**) EG-1, day 14; (**K**) EG-2, day 14; (**L**) EG-3, day 14; (**M**) CG-1, day 30; (**N**) CG-3, day 30. Hematoxylin-eosin. Magnification × 200.

Pooled semi-quantitative histological damage scores at 72 h are presented in Table 2. Typical histological changes in the ileum in the control and experimental groups on days 1, 14, and 30 are shown in Figure 2.

Irradiation without EACA administration (CG-3) was associated with the highest total mucosal damage score (29.0). All EACA administration regimens led to a significant reduction in damage severity compared with CG-3: in EG-1, the score was 24.0 (−17.2%; *p* = 0.008; in EG-2, it was 20.25 (−30.2%; *p* < 0.001); and in EG-3, it was 18.5 (−36.2%; *p* < 0.001). Among the active regimens, the combined scheme yielded the best results: in EG-3, the total score was lower than in EG-1 (*p* = 0.004) and EG-2 (*p* = 0.031).

Both non-irradiated controls differed markedly from CG-3 in the direction of less damage (CG-1: 7.0; CG-2: 7.7; both *p* < 0.001), while EACA administration without irradiation did not alter baseline morphology (CG-2 vs. CG-1: *p* = 0.41). Compared with the baseline control (CG-1), all irradiated groups, including those receiving EACA, retained higher damage scores (all *p* < 0.001); EG-3 scores also remained worse than in both non-irradiated groups (*p* < 0.001), indicating partial but incomplete histological protection, with maximal efficacy observed in the “before + after irradiation” regimen.

### 3.3. Villus Height

**Figure 3:**
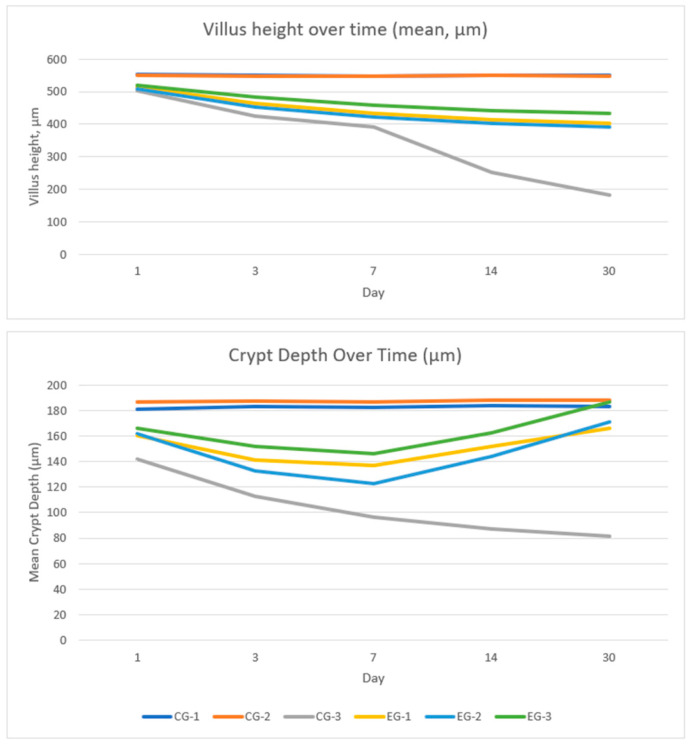
Longitudinal changes in ileal villus height and crypt depth.

The morphometric data on villus height (mean ± SD) with pairwise *p*-values adjusted by the Tukey–Holm method at each time point are presented in Figure 3, Table S1. At all timepoints, no differences were observed between the non-irradiated controls with and without EACA (CG-1 vs. CG-2; all *p* > 0.50), indicating that EACA does not affect villus height in the absence of irradiation.

On day 1, no significant differences were noted (CG-3 compared with any EACA regimen: *p* = 0.072, 0.11, 0.061, 0.083), i.e., early post-radiation changes were not distinguishable between irradiated groups. By day 3, irradiation had substantially reduced villus height in CG-3, and all EACA regimens showed significantly greater villus height compared with CG-3 (CG-3 vs. EG-1 *p* = 0.004; vs. EG-2 *p* < 0.001; vs. EG-3 *p* < 0.001). The same pattern persisted on day 7 (CG-3 vs. EG-1 *p* = 0.003; vs. EG-2 *p* < 0.001; vs. EG-3 *p* < 0.001) and became more pronounced on day 14 (all comparisons of CG-3 with EACA *p* < 0.001) and day 30 (all *p* < 0.001), indicating a sustained and increasing effect of EACA in preserving/restoring villus height after irradiation.

Despite this effect, EG-3 values remained significantly lower than those of the non-irradiated CG-1 from day 3 to day 14 (all *p* < 0.001) and were still moderately lower on day 30 (*p* = 0.006), indicating substantial but incomplete mucosal recovery by the end of the first month.

### 3.4. Crypt Depth

Morphometric data on intestinal crypt depth (µm) over the 1–30 day interval—presented as mean ± SD with pairwise *p*-values adjusted by the Tukey–Holm method—are shown in Figure 3, Table S2. At all timepoints, no significant differences were observed between the non-irradiated controls CG-1 and CG-2 (*p* ≥ 0.29), indicating that EACA by itself does not affect crypt depth.

In irradiated animals, CG-3 showed a substantially lower crypt depth compared with each of the EACA-treated groups (EG-1, EG-2, EG-3) at every time point from day 1 to day 30 (all *p* ≤ 0.018 on day 1 and *p* ≤ 0.009 on days 3–30), confirming a consistent radioprotective effect of EACA. By way of illustration: on day 7, the values were 96.5 ± 13.9 µm in CG-3 versus 136.7 ± 11.6, 122.4 ± 12.3, and 146.2 ± 10.8 µm in EG-1/EG-2/EG-3, respectively (in all CG-3 vs. EG comparisons *p* ≤ 0.006); on day 30, they were 81.3 ± 14.8 µm in CG-3 versus 166.2 ± 12.5, 171.0 ± 11.9, and 186.5 ± 12.1 µm, respectively (all *p* < 0.001).

In addition, EG-3 values remained significantly lower than those of CG-1 on days 1–14 (*p* = 0.018, 0.009, 0.006, 0.004), but by day 30 the difference disappeared (*p* = 0.27), indicating restoration of crypt depth to levels comparable with the non-irradiated controls.

### 3.5. Hemoglobin

**Figure 4:**
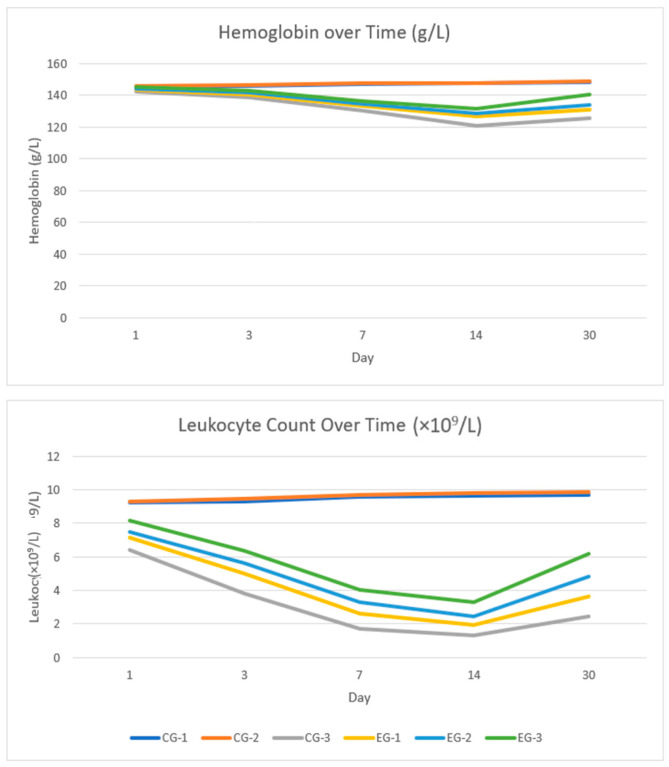
Longitudinal changes in hemoglobin and leukocytes.

Long-term 30-day hemoglobin trajectories (mean ± SD with pairwise *p*-values adjusted by the Tukey–Holm method) are presented in Figure 4, Table S3. At all timepoints, Hb levels in the non-irradiated groups CG-1 and CG-2 were virtually identical (*p* ≥ 0.59), confirming the absence of an independent effect of EACA in the absence of irradiation.

In the early period, no marked differences were observed between the irradiated control and the experimental EACA regimens: on day 1, hemoglobin levels were comparable across all irradiated groups. By day 3, Hb became noticeably higher in the groups receiving EACA after irradiation and in the combined regimen than in CG-3 (for example, 141.5–142.8 g/L in EG-2/EG-3 vs. 138.4 g/L in CG-3; *p* = 0.045 and *p* = 0.021, respectively), whereas administration only before irradiation (EG-1) had not yet produced a statistically significant increase.

Starting from day 7 and onward (days 14 and 30), hemoglobin levels were consistently higher in all three EACA regimens than in the irradiated control (in all CG-3 vs. EG comparisons *p* ≤ 0.024). The difference averaged several grams per liter and persisted until the end of the observation period, indicating a sustained protective effect of EACA against radiation-induced anemia.

At the same time, even in the most effective combined group EG-3, hemoglobin levels remained lower than in the baseline non-irradiated control CG-1 on days 7, 14, and 30 (*p* = 0.004, 0.002, and 0.001, respectively), although no differences were observed on days 1 and 3. This indicates partial but incomplete correction of anemia: EACA attenuates the Hb decline compared with the irradiated control but does not fully restore hemoglobin to normal levels by the end of the first month.

### 3.6. Leukocytes

The long-term dynamics of leukocyte counts (×10^9^/L) with pairwise *p*-values adjusted by the Tukey–Holm method are presented in Figure 4, Table S4. At all timepoints, the non-irradiated groups CG-1 and CG-2 showed similar and stable values (approximately 9.2–9.9 × 10^9^/L; all *p* ≥ 0.66), confirming the absence of an independent effect of EACA in the absence of irradiation.

Among irradiated animals, the most pronounced leukopenia developed in the untreated group (CG-3): by days 7–30, leukocyte counts had decreased to approximately 1.3–2.4 × 10^9^/L. Against the background of EACA, the decline was less severe. On day 1, all three experimental groups showed a tendency toward higher values compared with CG-3; however, a statistically significant difference was observed only in the combined regimen EG-3 (*p* = 0.032), whereas EG-1 and EG-2 differed from CG-3 only at the trend level (*p* = 0.12 and 0.09, respectively).

From day 3 through day 30, all EACA administration regimens (EG-1, EG-2, EG-3) provided significantly higher leukocyte counts compared with the irradiated control (in all CG-3 vs. EG comparisons *p* ≤ 0.024). The effect followed a consistent hierarchy of EG-3 > EG-2 > EG-1. For example, on day 7 the mean leukocyte count in CG-3 was 1.71 ± 0.69 × 10^9^/L, whereas in EG-1, EG-2, and EG-3 it was 2.61 ± 0.81, 3.28 ± 0.85, and 4.05 ± 0.87 × 10^9^/L, respectively.

Despite the marked attenuation of leukopenia with EACA, even in the most effective combined group EG-3 the values remained lower than in the non-irradiated control CG-1 on all observation days (*p* = 0.041 on day 1; *p* < 0.001 on days 3–30). This indicates partial but incomplete hematologic recovery by day 30.

### 3.7. Platelets

**Figure 5:**
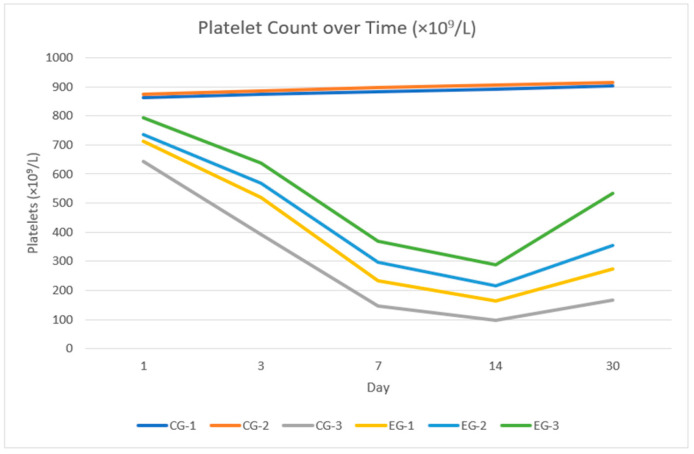
Longitudinal changes in platelets.

The long-term dynamics of platelet counts (×10^9^/L) with pairwise *p*-values adjusted by the Tukey–Holm method are presented in Figure 5, Table S5. In non-irradiated animals, EACA by itself did not affect the platelet compartment: CG-1 and CG-2 showed comparable levels at all time points (≈860–920 × 10^9^/L; all *p* ≥ 0.64).

After irradiation, a pronounced thrombocytopenia developed in the untreated group (CG-3), with platelet counts falling from ~640 × 10^9^/L on day 1 to ~150 × 10^9^/L on day 7 and ~100 × 10^9^/L on day 14. Against the background of EACA, the depth of this decline was substantially reduced and depended on the administration regimen. As early as day 1, platelet counts were noticeably higher than in CG-3 with post-irradiation and combined administration (EG-2 736.9 ± 55.1 and EG-3 792.4 ± 58.6 vs. 642.3 ± 49.8 × 10^9^/L in CG-3; *p* = 0.041 and *p* = 0.012, respectively), whereas the pre-irradiation-only regimen (EG-1 712.6 ± 53.9 × 10^9^/L; *p* = 0.083) showed only a non-significant trend.

By day 3, all three EACA regimens significantly outperformed CG-3, and this difference became more pronounced during the nadir period. For example, on day 7 the mean platelet count was 147.9 ± 36.8 × 10^9^/L in CG-3 versus 233.4 ± 45.3, 297.6 ± 49.8, and 369.8 ± 53.1 × 10^9^/L in EG-1, EG-2, and EG-3, respectively (in all CG-3 vs. EG comparisons *p* ≤ 0.004). On days 14 and 30, all EACA regimens continued to provide substantially higher values than the irradiated control (all *p* ≤ 0.031), with a consistent hierarchy of efficacy at all time points: EG-3 > EG-2 > EG-1.

At the same time, even in the most favorable combined group EG-3, platelet counts remained significantly lower than in the non-irradiated control CG-1 from day 1 onward (*p* = 0.023 on day 1; *p* ≤ 0.006 on days 3–30, most *p* < 0.001). This indicates that EACA markedly attenuates radiation-induced thrombocytopenia and accelerates recovery of the platelet compartment but does not achieve full normalization to the levels of non-irradiated animals even under the two-time-point regimen.

### 3.8. Kaplan–Meier Survival Analysis

**Figure 6:**
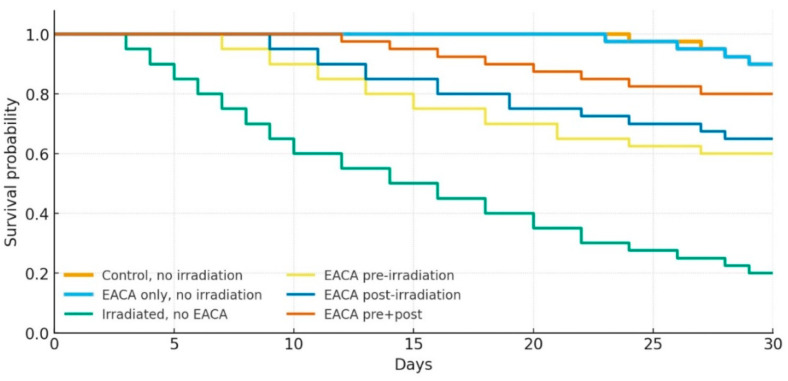
Survival of animal groups (the Kaplan–Meier method).

During the 30-day observation period, Kaplan–Meier survival analysis revealed marked differences between groups (log-rank χ^2^ = 26.4, *p* < 0.001; Figure 6). In both non-irradiated control groups (CG-1 and CG-2), survival at day 30 remained high at 90%. In contrast, irradiated animals without pharmacologic protection (CG-3) showed the poorest outcome, with only 20% surviving to day 30.

Administration of EACA improved survival in a regimen-dependent manner. Pre-irradiation EACA (EG-1) increased 30-day survival to 60% (*p* = 0.021 vs. CG-3), whereas post-irradiation treatment (EG-2) yielded a slightly better outcome (65%, *p* = 0.011 vs. CG-3). The combined regimen (EG-3, pre- and post-irradiation) produced the strongest protective effect, with 80% survival at day 30 (*p* < 0.001 vs. CG-3) and a clear rightward shift of the survival curve.

Pairwise log-rank tests confirmed significantly better survival in all EACA-treated irradiated groups compared with untreated irradiated controls, whereas no difference was detected between the two non-irradiated controls (CG-1 vs. CG-2, *p* = 0.74). Nonetheless, survival in EG-3 remained slightly lower than in CG-1 (*p* = 0.042), indicating partial but not complete restoration of resistance to radiation injury.

Table 3 summarizes the efficacy of EACA versus the irradiated control (CG-3) across all endpoints. RGS pain was reduced (OR: EG-1, 0.72; EG-2, 0.56; EG-3, 0.42), and the RIIMS_sum at 72 h decreased by 17%, 30%, and 36% in EG-1, EG-2, and EG-3, respectively. Villus height was higher in all EACA groups from day 3 onward, and crypt depth exceeded CG-3 at all timepoints, with EG-3 approximating CG-1 by day 30. Hemoglobin was higher from day 7 (earlier for EG-2/EG-3), while leukocytes and platelets were higher from day 3 (platelets already elevated on day 1 in EG-2/EG-3). Thirty-day survival improved from 20% in CG-3 to 60% (EG-1), 65% (EG-2), and 80% (EG-3), demonstrating a consistent efficacy gradient of EG-3 > EG-2 > EG-1.

## 4. Discussion

Our study demonstrated that ε-aminocaproic acid exerts a pronounced radioprotective effect in a model of acute total-body gamma irradiation in rats, affecting both the intestinal and hematopoietic systems. Administration of EACA was associated with reduced severity of radiation enteritis, attenuation of anemia, leukopenia, and thrombocytopenia, and improved 30-day survival, with the greatest effect observed under the combined regimen of pre- and post-irradiation administration. This hierarchy of regimens (EG-3 ≥ EG-2 > EG-1) was consistently reproduced across morphological, hematological, and clinical (pain, survival) outcomes, indicating a systemic pattern of protection rather than an isolated effect on a single organ.

The obtained results should be viewed in the context of the accumulated data on other experimental radioprotectors. The agents described in the literature form a heterogeneous group: these include small-molecule antioxidants and radiomodifiers (Ex-RAD^®^, octadecenyl thiophosphate, a combination of melatonin and metformin) [10,20,23], receptor-mediated modulators and immune agonists (the LPA2 receptor agonist DBIBB, the TLR5 agonist CBLB502, recombinant MFG-E8) [21,24,25], as well as cytokines and hematopoietic growth factors (primarily G-CSF) [13], which, in preclinical models, provide combined protection of the intestinal mucosa and bone marrow hematopoiesis, reducing mortality and the severity of the gastrointestinal and hematopoietic syndromes.

Against this background, ε-aminocaproic acid occupies a distinct niche as an inhibitor of fibrinolysis and proteolysis, and the reduction in intestinal injury and cytopenias observed in our study—comparable in direction and magnitude—allows EACA to be classified within the same group of multifactorial radioprotectors that act simultaneously on the gastrointestinal and hematopoietic components of acute radiation syndrome.

At the same time, our contribution differs fundamentally from previous studies on ε-aminocaproic acid. Previously, EACA was considered primarily as an antifibrinolytic agent with established clinical use in hemostatic therapy [26], and its radioprotective potential was investigated mainly in vitro and in selected models that did not comprehensively address both the intestine and hematopoiesis EACA has been shown to reduce the formation of reactive oxygen species and DNA damage in human blood cells, as well as to attenuate radiation-induced increases in capillary permeability; however, there have been no systematic in vivo data on preservation of the crypt compartment, the dynamics of hematologic parameters, and survival under high-dose total-body irradiation. In this context, our study is essentially the first integrated description of the radioprotective effects of EACA simultaneously at the level of the intestinal mucosa, bone marrow hematopoiesis, and clinical outcomes in a model of severe acute radiation syndrome.

When interpreting these results, it is important to consider the potential mechanisms of EACA action. From a classical standpoint, it is a lysine analogue with an antifibrinolytic effect due to competitive blockade of lysine-binding sites on plasminogen and plasmin [27]. This leads to a reduction in pericellular fibrinolysis and plasmin-mediated degradation of extracellular matrix components [28]. In the setting of radiation-induced intestinal injury, such an effect may limit secondary protease-dependent stromal damage, edema, and necrosis, thereby creating more favorable conditions for the survival of crypt stem cells and epithelial repair.

In parallel, the literature provides evidence that EACA can modulate oxidative stress by reducing ROS levels and DNA damage [18,19], which is consistent with the concept of a key role of ROS and redox regulation (Nrf2, NF-κB) in radiation injury to the small intestine and bone marrow [29]. Although these pathways were not directly examined in our work, the combined morphological and hematologic effects make it plausible to hypothesize that a combination of protease and redox modulation underlies the radioprotective action of EACA.

Our data also highlight the importance of the administration regimen. The marked advantage of the combined scheme (before + after irradiation) over monotherapy given either before or after exposure is consistent with the concept that a radioprotector should be present both during the phase of the primary radiation insult and during the subsequent phases of inflammation and repair. A similar dependence on dosing regimen has previously been reported for other radioprotectors, including combinations of antioxidants and cytokines [30,31]. In the case of EACA, this may reflect the need to suppress both early protease- and ROS-mediated events and later cascades of matrix remodeling and inflammation.

### Limitations

Our study has several limitations. First, we did not perform direct molecular measurements of plasmin system activity, oxidative stress markers, or the expression of key regulatory pathways (Nrf2, NF-κB, caspases); therefore, the mechanisms discussed remain hypothetical and require confirmation in targeted experimental studies. Second, we used a single fixed irradiation dose (10.6 Gy) and a single dose of ε-aminocaproic acid, which does not allow assessment of dose–response relationships or the potential therapeutic window. Third, the protocol predominantly reflects the acute period (30 days); the potential impact of ε-aminocaproic acid on late radiation complications (fibrosis, vascular changes) was not evaluated in this study.

Fourth, the use of healthy young rats and an acute total-body irradiation model does not recapitulate key clinical features of radiotherapy and oncology settings, including localized fractionated exposure and disease- or treatment-related alterations in hemostasis and inflammation. Consequently, direct extrapolation to patients—particularly those with cancer and comorbidities—should be made with caution. As a next step, tumor-bearing models with fractionated, localized irradiation (± chemotherapy) will be required to confirm that EACA does not compromise tumor control and to define safety/benefit in clinically relevant contexts.

Finally, quantitative parameters were analyzed separately for each time point without accounting for within-subject correlation of repeated measurements, and the time points for euthanasia were pre-specified by the protocol and did not depend on the animals’ clinical status; as a result, the histological data reflect fixed time snapshots, and the longitudinal dynamics of mucosal injury and repair may not have been fully captured.

Despite these limitations, our results demonstrate that ε-aminocaproic acid has a multifactorial radioprotective potential in a model of severe acute radiation syndrome, with maximal efficacy achieved when it is administered both before and after irradiation. The combination of EACA’s well-established safety profile as a hemostatic agent with the intestinal and hematologic effects identified in our study makes it an attractive candidate for further preclinical investigation across a broader range of doses and models and, in the longer term, for the design of early clinical trials in populations at high risk of radiation injury.

## 5. Conclusions

The present study demonstrated that ε-aminocaproic acid has a notable radioprotective potential in a rat model of acute total-body gamma irradiation (10.6 Gy), attenuating the severity of radiation enteritis, reducing the extent of hematologic disturbances, and improving 30-day survival, with the greatest effect observed under the combined regimen of administration before and after irradiation. The integrated improvement in morphological, hematologic, and clinical parameters, while residual damage persists, supports consideration of EACA as a promising candidate for further preclinical studies across a wider range of doses and administration regimens, followed by evaluation of its potential for clinical translation.

## Figures and Tables

**Table 1:** Odds ratios for higher Rat Grimace Scale pain category in EACA-treated rats compared with irradiated controls (CG-3) across the 30-day observation period.

Comparison (vs. CG-3)	Odds Ratio for Higher Pain Category	95% CI	*p*-Value
EG-1 (EACA −3 h) vs. CG-3	0.72	0.55–0.94	0.016
EG-2 (EACA +3 h) vs. CG-3	0.56	0.41–0.73	<0.001
EG-3 (EACA −3 h/+3 h) vs. CG-3	0.42	0.31–0.58	<0.001

**Table 2:** Overall semi-quantitative histological injury score 72 h after irradiation and pairwise comparisons between groups.

GROUP	Overall Injury Score	Reduction vs. CG-3, %	*p* vs. CG-3	*p* vs. CG-1	*p* vs. EG-3
CG-1 (no irradiation/no EACA)	7.0	–	<0.001	–	<0.001
CG-2 (EACA/no irradiation)	7.7	–	<0.001	0.41	<0.001
CG-3 (irradiation/no EACA)	29.0	0	–	<0.001	<0.001
EG-1 (EACA 3 h before)	24.0	17.2	0.008	<0.001	0.004
EG-2 (EACA 3 h after)	20.25	30.2	<0.001	<0.001	0.031
EG-3 (EACA 3 h before and 3 h after)	18.5	36.2	<0.001	<0.001	–
Kruskal–Wallis					

**Table 3:** Unified summary of endpoints and EACA effects.

Endpoint	Readout/Metric	Summary vs. CG-3
RGS pain	OR vs. CG-3	EG-1: 0.72; EG-2: 0.56; EG-3: 0.42
RIIMS_sum (72 h)	% change vs. CG-3	EG-1: −17%; EG-2: −30%; EG-3: −36%
Villus height	Between-group difference over time	All EG > CG-3 from day 3
Crypt depth	Between-group difference over time	All EG > CG-3 at all timepoints
Hemoglobin	Longitudinal (days 1–30)	All EG > CG-3 from day 7 (earlier for EG-2/EG-3)
Leukocytes	Longitudinal (days 1–30)	All EG > CG-3 from day 3
Platelets	Longitudinal (days 1–30)	All EG > CG-3 from day 3 (EG-2/EG-3 higher already day 1)
Survival (day 30)	% alive	CG-3: 20% vs. EG-1: 60%, EG-2: 65%, EG-3: 80%

## Data Availability

The original contributions presented in this study are included in the article/Supplementary Materials. Further inquiries can be directed to the corresponding author.

## Supplementary Materials

The following supporting information can be downloaded at: https://www.mdpi.com/article/10.3390/life16010096/s1, Table S1: Morphometric indicators of villus height (µm) in rat ileum across groups and time points (mean ± SD and Tukey adjusted *p*-values); Table S2: Intestinal crypt depth (µm) and pairwise significance at days 1–30 post-irradiation (mean ± SD, Tukey adjusted *p*-values); Table S3: Longitudinal changes in hemoglobin (g/L) across six rat groups over 30 days post-irradiation (mean ± SD and Tukey adjusted *p*-values); Table S4: Longitudinal changes in leukocyte count (×10^9^/L) across six rat groups over 30 days post-irradiation; Table S5: Longitudinal changes in platelet count (×10^9^/L) across six rat groups over 30 days post-irradiation.
