# Supplementary material for: Radioprotective Effect of ε-Aminocaproic Acid in Acute Total-Body Gamma Irradiation in Rats
Source: Life (Basel). 2026 Jan 8;16(1):96. doi: 10.3390/life16010096 (PMC12843070; doi:10.3390/life16010096)
Supplement: Supplementary file 1 [file life-16-00096-s001.zip › life-4056834-supplementary.pdf]

Supplementary Materials

Table S1. Morphometric indicators of villus height (μm) in rat ileum across groups and time points (mean ± SD and Tukey adjusted p-values).

Day	GROUP									
	CG-1	CG-2	*p (CG-1 vs CG-2)*	CG-3	*p (CG-3 vs EG-1)*	EG-1	*p (CG-3 vs EG-2)*	EG-2	*p (CG-3 vs EG-3)*	EG-3	*p (EG-3 vs CG-1)*
1	552.3 ± 13.4	549.8 ± 12.1	0.64	501.6 ± 16.9	0.072	512.7 ± 14.8	0.11	507.9 ± 15.6	0.061	518.5 ± 13.9	0.083
3	549.1 ± 17.5	546.7 ± 13.2	0.58	423.4 ± 24.2	0.004	462.9 ± 18.1	0.001	452.7 ± 17.6	<0.001	482.3 ± 19.4	<0.001
7	548.6 ± 14.3	547.2 ± 13.7	0.71	392.5 ± 21.3	0.003	433.8 ± 16.9	0.001	423.1 ± 18.4	<0.001	457.9 ± 17.2	<0.001
14	550.4 ± 13.1	551.6 ± 12.9	0.82	252.7 ± 19.1	<0.001	412.5 ± 15.8	<0.001	402.3 ± 16.2	<0.001	442.1 ± 18.0	<0.001
30	551.2 ± 12.7	548.9 ± 13.3	0.69	181.9 ± 17.2	<0.001	401.6 ± 14.7	<0.001	391.4 ± 15.1	<0.001	432.8 ± 15.5	0.006
Statistical analysis was performed at each time point using one-way ANOVA followed by Tukey adjusted post-hoc tests											

Table S2. Intestinal crypt depth (μm) and pairwise significance at days 1–30 post-irradiation (mean ± SD, Tukey adjusted p-values).

Day	GROUP										
	CG-1	CG-2	*P (CG-1 vs CG-2)*	CG-3	*p (CG-3 vs EG-1)*	EG-1	*p (CG-3 vs EG-2)*	EG-2	*p (CG-3 vs EG-3)*	EG-3	*p (EG-3 vs CG-1)*
1	181.3 ± 10.7	186.5 ± 9.9	0.41	141.6 ± 11.8	0.006	160.4 ± 11.2	0.004	162.1 ± 11.5	0.002	165.8 ± 10.6	0.018
3	183.0 ± 10.2	187.6 ± 10.7	0.37	112.4 ± 14.7	0.001	141.3 ± 12.8	<0.001	132.7 ± 12.1	<0.001	151.9 ± 11.3	0.009
7	182.2 ± 9.8	186.9 ± 10.1	0.33	96.5 ± 13.9	<0.001	136.7 ± 11.6	<0.001	122.4 ± 12.3	<0.001	146.2 ± 10.8	0.006
14	184.1 ± 10.3	187.8 ± 9.7	0.35	86.9 ± 15.1	<0.001	151.5 ± 12.2	<0.001	143.8 ± 11.5	<0.001	162.7 ± 11.0	0.004
30	183.4 ± 10.6	188.2 ± 9.8	0.29	81.3 ± 14.8	<0.001	166.2 ± 12.5	<0.001	171.0 ± 11.9	<0.001	186.5 ± 12.1	0.27
Statistical analysis was performed at each time point using one-way ANOVA followed by Tukey adjusted post-hoc tests											

Table S3. Longitudinal changes in hemoglobin (g/L) across six rat groups over 30 days post-irradiation (mean ± SD and Tukey adjusted p-values).

Day	GROUP										
	CG-1	CG-2	*p (CG-1 vs CG-2)*	CG-3	*p (CG-3 vs EG-1)*	EG-1	*p (CG-3 vs EG-2)*	EG-2	*p (CG-3 vs EG-3)*	EG-3	*p (EG-3 vs CG-1)*
1	145.2 ± 4.3	145.7 ± 4.1	0.68	142.1 ± 6.5	0.21	143.5 ± 5.4	0.18	144.2 ± 5.3	0.12	145.1 ± 5.0	0.79
3	146.0 ± 4.4	146.3 ± 4.2	0.73	138.4 ± 7.2	0.081	140.6 ± 6.5	0.045	141.5 ± 6.4	0.021	142.8 ± 5.3	0.064

Day	GROUP									
	CG-1	CG-2	*p (CG-1 vs CG-2)*	CG-3	*p (CG-3 vs EG-1)*	EG-1	*p (CG-3 vs EG-2)*	EG-2	*p (CG-3 vs EG-3)*	EG-3	*p (EG-3 vs CG-1)*
7	147.1 ± 4.5	147.4 ± 4.4	0.70	130.2 ± 8.3	0.024	133.2 ± 6.7	0.013	134.3 ± 6.6	0.006	136.4 ± 5.4	0.004
14	147.5 ± 4.8	147.9 ± 4.9	0.65	120.6 ± 9.0	0.019	126.8 ± 6.9	0.006	128.7 ± 6.8	<0.001	131.5 ± 5.6	0.002
30	148.3 ± 4.6	148.7 ± 4.7	0.59	125.4 ± 8.6	0.011	130.7 ± 6.7	0.002	133.6 ± 6.6	<0.001	140.2 ± 5.3	0.001
Statistical analysis was performed at each time point using one-way ANOVA followed by Tukey adjusted post-hoc tests											

Table S4. Longitudinal changes in leukocyte count (×10^9/L) across six rat groups over 30 days post-irradiation.

Day	GROUP										
	CG-1	CG-2	*p (CG-1 vs CG-2)*	CG-3	*p (CG-3 vs EG-1)*	EG-1	*p (CG-3 vs EG-2)*	EG-2	*p (CG-3 vs EG-3)*	EG-3	*p (EG-3 vs CG-1)*
1	9.24 ± 1.63	9.31 ± 0.82	0.71	6.41 ± 1.25	0.12	7.12 ± 1.54	0.09	7.46 ± 0.98	0.032	8.19 ± 0.91	0.041
3	9.30 ± 0.89	9.45 ± 0.77	0.68	3.78 ± 1.19	0.020	5.01 ± 1.07	0.006	5.63 ± 1.02	0.001	6.38 ± 1.51	<0.001
7	9.56 ± 0.92	9.71 ± 0.68	0.66	1.71 ± 0.69	0.004	2.61 ± 0.81	0.001	3.28 ± 0.85	<0.001	4.05 ± 0.87	<0.001
14	9.63 ± 0.74	9.79 ± 1.23	0.69	1.29 ± 0.43	0.024	1.93 ± 0.76	0.004	2.47 ± 0.72	<0.001	3.32 ± 0.99	<0.001
30	9.71 ± 0.68	9.86 ± 1.95	0.73	2.43 ± 1.07	0.018	3.64 ± 0.97	<0.001	4.82 ± 1.02	<0.001	6.21 ± 1.34	<0.001

Table S5. Longitudinal changes in platelet count (×10^9/L) across six rat groups over 30 days post-irradiation.

Day	GROUP										
	CG-1	CG-2	*p (CG-1 vs CG-2)*	CG-3	*p (CG-3 vs EG-1)*	EG-1	*p (CG-3 vs EG-2)*	EG-2	*p (CG-3 vs EG-3)*	EG-3	*p (EG-3 vs CG-1)*
1	862.7 ± 55.2	874.1 ± 62.8	0.64	642.3 ± 49.8	0.083	712.6 ± 53.9	0.041	736.9 ± 55.1	0.012	792.4 ± 58.6	0.023
3	872.9 ± 56.1	884.7 ± 60.2	0.67	392.5 ± 43.7	0.014	517.8 ± 50.7	0.004	568.3 ± 51.9	0.001	637.1 ± 54.2	0.006
7	883.6 ± 54.4	896.2 ± 58.5	0.70	147.9 ± 36.8	0.004	233.4 ± 45.3	0.001	297.6 ± 49.8	<0.001	369.8 ± 53.1	<0.001
14	892.8 ± 51.9	905.3 ± 57.6	0.72	96.7 ± 33.4	0.031	162.9 ± 41.8	0.003	214.5 ± 46.1	<0.001	287.9 ± 52.3	<0.001
30	903.5 ± 57.8	915.9 ± 60.7	0.69	167.4 ± 39.9	0.027	272.6 ± 48.9	<0.001	354.1 ± 53.8	<0.001	532.6 ± 60.5	<0.001
